# Deterministic culturing of single cells in 3D

**DOI:** 10.1038/s41598-020-67674-3

**Published:** 2020-07-02

**Authors:** Rohil Jain, Shirisha Chittiboyina, Chun-Li Chang, Sophie A. Lelièvre, Cagri A. Savran

**Affiliations:** 1 School of Mechanical Engineering, Purdue University, West Lafayette, IN 47907 USA; 2 Birck Nanotechnology Center, Purdue University, West Lafayette, IN 47907 USA; 3 Weldon School of Biomedical Engineering, Purdue University, West Lafayette, IN 47907 USA; 4 Department of Basic Medical Sciences, College of Veterinary Medicine, Purdue University, West Lafayette, IN 47907 USA; 5 Center for Cancer Research, Purdue University, West Lafayette, IN 47907 USA

**Keywords:** Cell culture, Tissue culture, Biomedical engineering, Tumour heterogeneity, Cancer models

## Abstract

Models using 3D cell culture techniques are increasingly accepted as the most biofidelic in vitro representations of tissues for research. These models are generated using biomatrices and bulk populations of cells derived from tissues or cell lines. We present an alternate method to culture individually selected cells in relative isolation from the rest of the population under physiologically relevant matrix conditions. Matrix gel islands are spotted on a cell culture dish to act as support for receiving and culturing individual single cells; a glass capillary-based microfluidic setup is used to extract each desired single cell from a population and seed it on top of an island. Using examples of breast and colorectal cancers, we show that individual cells evolve into tumors or aspects of tumors displaying different characteristics of the initial cancer type and aggressiveness. By implementing a morphometry assay with luminal A breast cancer, we demonstrate the potential of the proposed approach to study phenotypic heterogeneity. Results reveal that intertumor heterogeneity increases with time in culture and that varying degrees of intratumor heterogeneity may originate from individually seeded cells. Moreover, we observe that a positive relationship exists between fast growing tumors and the size and heterogeneity of their nuclei.

## Introduction

Three-dimensional (3D) cell culture methods are increasingly used to generate complex tissue models. Multicellular structures created by 3D cell culture should mimic aspects of in vivo microenvironments and generate organized cell assemblies that are biologically, histologically and molecularly more similar to in vivo conditions than standard 2D cultures^1^. Such models developed with cancer cells also constitute an ideal platform for in vitro testing of therapeutic drugs^1, 2^. Cell lines and primary cells from patients’ cancerous tissues have been successfully used in 3D cell cultures^3^ to produce tumors (which we define as abnormal growths of tissue).

Methods that employ non-adherent conditions including the hanging drop method^4^, rotating bioreactor^5, 6^, magnetic levitation^7^ or microfabricated modalities in various forms^8, 9^ have been reported. Some of the most widely used non-adherent techniques do not represent a true 3D cell culture that mimics tumor formation in vivo. When tens-of-thousands cells are aggregated into a spheroid (i.e., a mass with spherical shape) such as in a hanging drop, reactor or U-bottom plates, an extensive central necrotic core forms over a few hours due to the lack of nutrient and oxygen penetration beyond a 200 µm depth. Extended central necrosis is a rare phenomenon in real cancers. This nonphysiologically-relevant cancer representation is exacerbated by the lack of progressive tumor development via cell division and the lack of interaction with an appropriate extracellular matrix (ECM).

Under adherent conditions, in the presence of a matrix, 3D cell culture can be achieved in simple culture vessels or within microfluidic devices that permit controlled supply of growth factors, drugs and other stimulants^10, 11^. Adherent 3D cell cultures may use specially designed matrices that mimic the porosity, stiffness, and adhesion strength of the original tissue^12^. Most 3D cell culture models that generate tumors, start with a large bulk of cells that is used to “seed” the culture vessel. Although cells in a seed may originate from the same population, they can still be phenotypically different from each other at the single cell level. Phenotypic variability exists in vivo where it creates hurdles in designing effective therapies (e.g. for cancer), requiring a better understanding of cellular heterogeneity^13–15^. The combination of subtle genetic variations and epigenetic traits arising from different sources of origin or microenvironmental conditions underlies phenotypic heterogeneity, since it leads to different protein expression patterns. To better understand cell-to-cell variations, tumor cells have been isolated from tumor tissue or bodily fluids and analyzed^16, 17^. Advances in sequencing techniques have helped tremendously the study of cell heterogeneity from a genomic perspective^18–20^. To further facilitate the study of cell heterogeneity from a functional perspective, it is highly desirable to generate tumor models that each originates from one cell. Such studies can elucidate heterogeneity within a tumor generated by the proliferation of one given cell, as well as the heterogeneity among tumors obtained from different single cells. This approach can, in turn, enable the quantitative measurement of phenotypic variability caused by the microenvironment as well as variability that is intrinsic to a given population of cells.

Automated technologies to separate a large number of cells into single cells of interest, such as Fluorescence Activated Cell Sorting, have been employed to dispense single cells into microwells for culture^21^. A limiting dilution method corresponding to the serial dilution of a suspension of cells has also been used to statistically (but not deterministically) contain one cell in a unit volume. This limiting dilution suspension is either mixed with an appropriate ECM or overlaid on top of it for cell culture^22–24^. While these methods are valid to obtain single cells from a large population of cells, they are impractical when the cell population is small as they generate considerable cell loss during mixing and/or transfer.

Microfluidic platforms have been developed to address the unique problem of single cell culture in 3D^25–29^. Among the examples, a micro-raft array was used to generate organoids by exposing a group of cells to the culture platform until each cell in the group randomly occupied a micro-raft^28^. Then, all micro-rafts were covered with a gel matrix (MATRIGEL). Also, a gel-island chip was used for the formation and the analysis of tumors that originate from single cancer stem-like cells^29^. In this method, a liquid suspension of cells embedded in a collagen matrix was flowed through the chip. The gel-encapsulated cells randomly occupied the islands, 34% of which ended up with a single cell. Then, the culture medium and growth factors were supplied through a channel to which all the islands were connected. A common trait of these platforms is that they generally start with a suspension containing a large number of cells that are ‘individualized’ by random confinement in microstructures. As a result, all cells in the original suspension are eventually cultured without discrimination. Even in cases that involve heterogenous mixtures of cells, where one may be interested in culturing and analyzing only a subset, all cells in the original mixtures need to be cultured and possibly analyzed. This approach requires fabricating many microstructures or wells so that each cell in the original suspension is accommodated. It also requires imaging of all microstructures in order to not miss the tumors originating from specific single cells, the proliferation of which is of interest. Moreover, the stochastic distribution of cells into the microstructures/wells introduces the possibility of multiple cells occupying a single well, which prevents developing a tumor from a single cell. Another commonality of current schemes is the confinement of single cells within a microstructure that introduces the possibility of altering the cell phenotype by mechanical restriction^30, 31^, as well as difficulties to extract individual tumors after the culture is completed. Especially in applications that involve rare cells^32^, such as circulating tumor cells (CTCs)^33–35^, metastasis initiating cells (MICs)^36–38^, or fetal cells^39, 40^, where the targeted cells are only a few and sometimes only one, a deterministic method that enables growing a tumor or another tissue only from a single targeted seed cell, as well as facile extraction of the resulting tissue for downstream analysis, would be highly advantageous.

**Figure 1:**
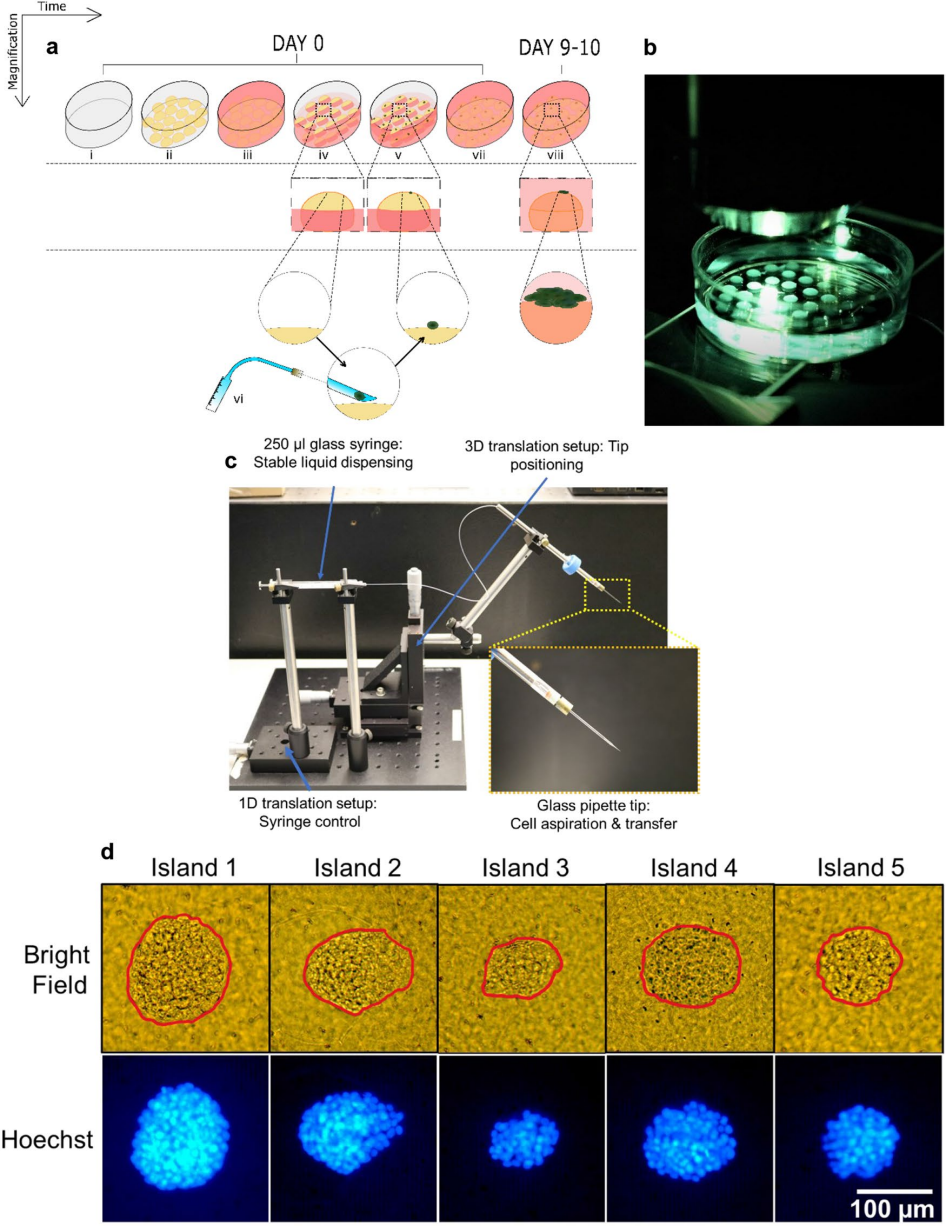
Individualized MCF7 single cell seeding on collagen I islands leads to tumor formation. **a** Steps followed to achieve single cell culture on collagen I islands: (i–iii) Islands of collagen matrix are formed on a culture dish and soaked in culture medium. (iv–vi) Each island is seeded with a single cell while the islands are partially submerged in culture medium. (vii) After cell adhesion, the dish is filled with culture medium to completely submerge the islands. (viii) Islands are incubated in culture medium for 9–10 days to allow single cells to form tumors. **b** Hemispherical islands (n = 40) of collagen I matrix on a polystyrene culture dish, where each island will receive exactly one single cell for culture. **c** Microfluidic setup assembled in-house for deterministic single cell picking and placement, with an inset that shows a zoomed in portion of the setup with focus on the glass tip. **d** Tumors developed from single MCF-7 cells, visualized using bright-field microscopy as well as Hoechst-based fluorescence microscopy after 10 days of culture (tumors are outlined in red using Microsoft EXCEL).

Here we describe a method (Fig. 1a–c) that uses a deterministically targeted single cell as the seed. Only the chosen single cells are individualized on top of collagen islands. Our approach combines the flexibility to choose single cells of interest through micromanipulation with the benefits of 3D culturing in physiological conditions, and explores the evolution of single cell-derived tumors. The tumors can be easily extracted from their culture location and transferred elsewhere for long-term culture or subsequent downstream analyses. Using morphometric parameters as an example, we demonstrate the method’s potential for the study of inter- and intratumor heterogeneity and show preliminary information regarding a potential relationship between tumor architecture and the organization of cell nuclei as it relates to cancer aggressiveness.

## Results

### Single cell culture at registered locations on collagen I islands reveals survival, proliferation and tumor formation potentials

We initially performed characterization experiments under standard 2D cell culture conditions to identify a cancer cell line with which to develop and test the deterministic 3D culturing method. Ten to twenty wells of a 96 well plate received one cell from breast (MCF-7), cervical (KB) and pancreatic cancer (LNCaP) cell lines in culture medium including 10% fetal bovine serum. Approximately 100 cells were seeded in a single well of the same 96 well plate from the same populations of cells as control. At least 50% of the single cells after seven to eight days of seeding had led to a small colony of cells (for all cell lines tested; Supplementary Fig. S1). The MCF-7 cell line was selected to pursue the development of the single cell 3D culture method in light of their high survival rate (95%). These cells are classified as luminal A type of breast cancer cells (estrogen receptor and progesterone receptor positive). This subtype of breast cancer is mostly nonaggressive, and in 3D cell culture MCF7 cells typically form tumors that rarely present invasive extensions or migratory patterns.

Collagen I was used as the ECM for 3D cell culture because it is the most abundant fibrous molecule surrounding tumors of epithelial origin like carcinomas, and it can be tuned to a desired stiffness to mimic that of cancerous tissues. Conventional matrix-based 3D cultures in standard well plates use a large amount of collagen spread over the area of each well. For standard 3D cell culture applications that use a large number of seed cells per unit volume of the collagen, it is possible to easily identify and image cells or clusters of cells of interest. However, when seeding a small number of single cells, it is challenging to find and image one particular cell that is almost transparent against a large pool of collagen matrix. This challenge is further exacerbated if the seeding location is not referenced, or the cell in question migrates and/or if it is embedded inside the matrix. Microplates, such as 384 and 1,056 well plates have smaller wells, which reduces the amount of collagen needed. However, this setting also reduces the amount of culture medium, i.e. nutrients that need to be supplied to the growing tissue. The necessary frequent changes of the culture medium are greatly impeded by the narrow space in these wells, that prevent the user from easily maneuvering a micropipettor. Therefore, we sought to design a method that has an optimal balance of collagen matrix and volume of medium to sustain the growth of tumors from single cells initially deposited in registered locations and enables an easy observation of the growth process.

We opted for small droplets (5 µl) of tunable Methacrylated Type I collagen matrix (referred to as collagen throughout the manuscript) with a Young’s modulus of 3,320 Pa (representative of increased ECM stiffness measured in invasive ductal carcinomas of the breast^41^) placed in a 35 mm polystyrene dish not treated for cell culture (Fig. 1a). Up to 50 droplets of matrix were deposited in the dish to form semi-ellipsoidal islands (Fig. 1b), each with a diameter of approximately 3 mm. Single cells were seeded on the islands using a cell picking setup developed in house (Fig. 1c). Each dish was incubated for 9–10 days at 37 °C, 5% CO_2_ and 95% relative humidity (see full description of the process in the Methods section). Culturing the cells on relatively small fixed spots referred to as ‘islands,’ allowed simple tracing of a particular cell by recording still images at various intervals, without continuous time-resolved microscopy that would otherwise be necessary if multiple cells were used (since cells can often migrate over small distances on the surface of collagen).

Once a cell is seeded on top of a collagen matrix island, there are two possible immediate outcomes; the cell may successfully bind to the collagen gel or it may not bind and float away. If the cell binds to collagen, it may or may not survive, and if it survives, it may (epi)genetically be predisposed to proliferate or find the culture conditions unsuitable for proliferation. To study the reproducibility of these outcomes, three biological replicates of the cultures (i.e., using three different batches of MCF7 cells) were performed. Collagen matrix islands were spotted on 35 mm dishes with the number of islands in each dish varying between 25 and 50. Each island was seeded with exactly one MCF-7 cell on Day 0 and observed on Day 1–2 to check for cell attachment and again on Day 9–10 to check for sustained attachment and proliferation under an upright microscope in brightfield mode. Hoechst staining was used to visualize cells in the tumor using fluorescence microscopy (Fig. 1d). As shown in Table 1, a great majority of the islands (83–98%) successfully retained a single MCF7 cell (the first column is the total number of islands in each dish that were seeded with a single MCF-7 cell on Day 0; the second column shows the percentage of islands each having a single cell successfully attached after one to two days). As illustrated in the third column of Table 1, the percentage of islands with visible cells 9–10 days after seeding was lower than at day 1; it might be due to cell death in the first few days of culture or detachment of the cells. The cell clusters that were visible on the islands at the end of the observation period varied in size. In summary, over the three biological replicates, 81–94% of the islands had visible cells (nonproliferating or organized into tumors; column 3 of Table 1) and 66–81% of the islands harbored tumors larger than 5,000 µm^2^ (column 4 of Table 1), indicating that the cells that seeded these tumors had a strong proliferation potential. From this first set of observations, it could also be concluded that individual cells that came from the same nominal (MCF7) population displayed different potentials to survive and proliferate to form tumors.

**Table 1:** Cell survival and proliferation efficiency: percentage of total number of islands.

	Total number of islands seeded (100%)	Islands with a cell on day 1–2 (%)	Islands with visible cells on day 9–10(%)	Islands with tumors of area > 0.005 mm^2^ on day 9–10 (%)
Exp. 1	26	96	92	77
Exp. 2	48	98	94	81
Exp. 3	47	83	81	66

### Single cell-derived tumors are reminiscent of the cancer type and reproduce intertumor heterogeneity

**Figure 2:**
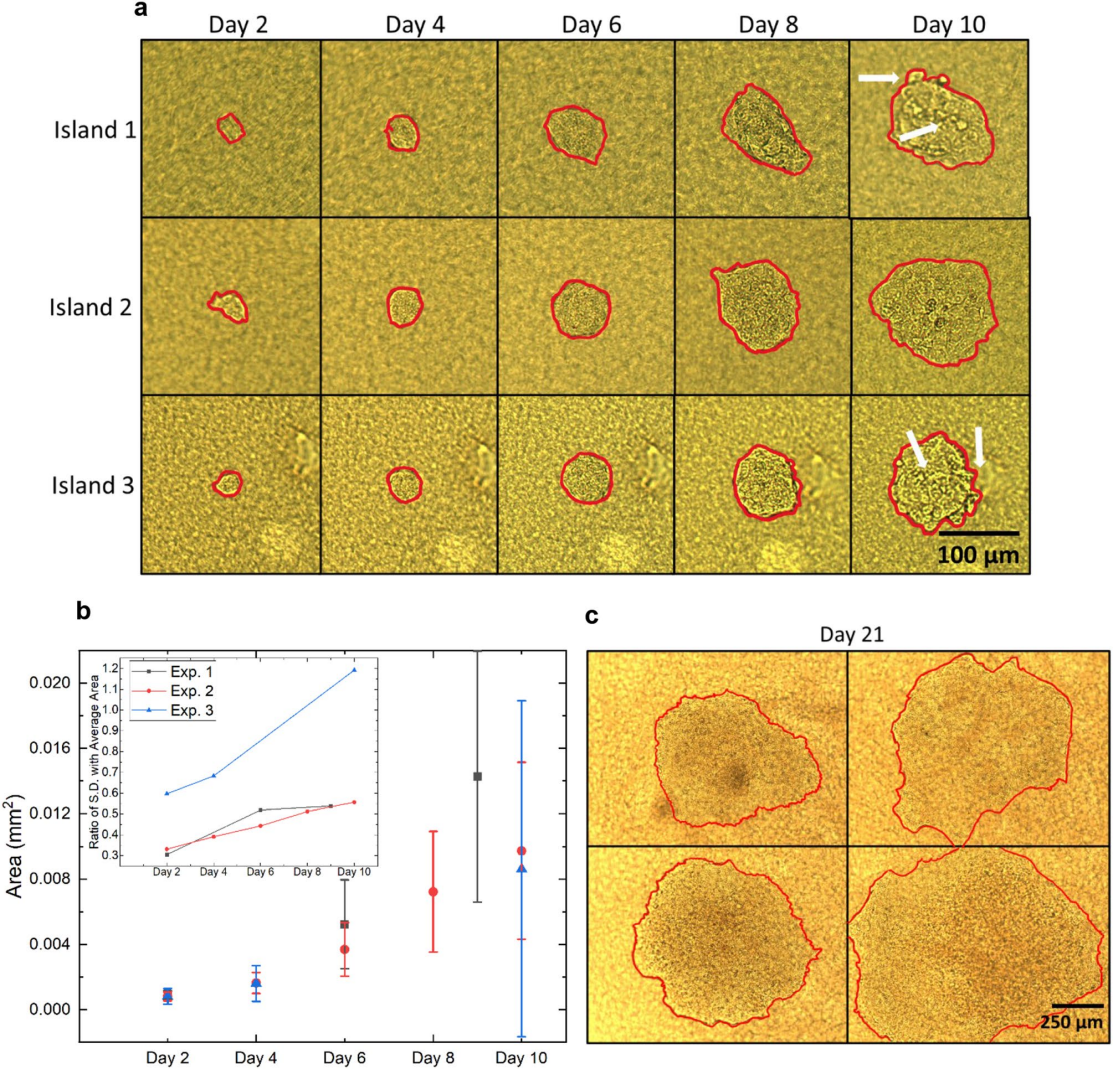
Island tracking over time reveals variable increases in tumor size. Single MCF7 cells were cultured on collagen I islands, as described in Fig. 1 and resulting tumors were manually outlined (as shown in red) to measure their areas with Image J. **a** Bright-field images of three tumors (Islands 1, 2, 3) over time. Grape-like features on the tumor top and at the periphery are indicated by white arrows. **b** Temporal evolution of tumor area over 10 days for three biological replicates. Each solid dot represents the average area of 26 (in black), 48 (in red) and 47 (in blue) tumors with standard deviation. Inset shows the increasing trend in ratio of standard deviation to the average of tumor area for each experiment over time. **c** Images of tumors from another experiment that were maintained in culture for 21 days.

An advantage of the current scheme is the inherent efficacy in tracking the behavior of a single cell at the beginning of the culture period. Locating and recognizing the cell on the collagen island is a rapid process, even if the cell migrates over small distances. Similarly, changes in the appearance of multicellular clusters and tumors can be made using bright-field upright microscopy. Hence, the cells can be observed by a sequence of still images, (as opposed to monitoring multiple cells using continuous time-resolved microscopy) as shown by multiple examples of a single MCF-7 cell evolving into a tumor (Fig. 2a–c). Islands 1 and 3, for instance show grape-like features on the tumor top and at the periphery, while for the tumor on island 2 the top surface appears regular or smooth (Fig. 2a, Supplementary Fig. S2 without markings). All experiments show an increase in tumor size based on the duration of culture, but with increasing disparity among tumors (Fig. 2b). In cultures kept for 20–25 days, some tumors continue to grow and cover most of the collagen island’s top surface, with a diameter above 500 µm (Fig. 2c). Very few tumors (on average two out of 30) display a dark central region. When observed under fluorescence, these regions are faintly colored in red after treatment with Propidium Iodide, confirming necrosis (Supplementary Fig. S3)^42^. Thus, cultures should be stopped before tumors reach 400 µm in size since the maximum penetration depth of nutrients and oxygen is ~ 200 µm. All analyses for the rest of the experiments were performed with 10–14 days old 3D cultures and tumors below 400 µm.

**Figure 3:**
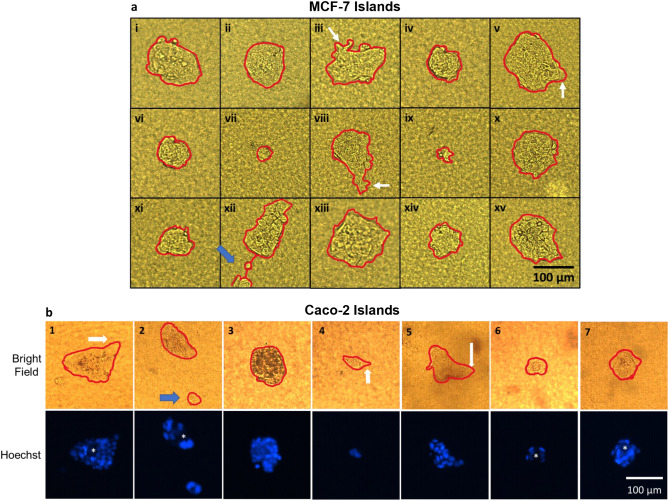
Tumors formed by single MCF7 and Caco-2 cells are heterogeneous. Individual MCF7 cells or individual Caco-2 cells were cultured on collagen I islands, as described in Fig. 1 and resulting tumors at day 10 of culture were outlined in red to visualize their size and shape. **a**: **i**–**xv** Bright-field images of MCF-7 tumors; short ‘arm-like’ structures are indicated by white arrows in iii and viii. **b**: **1**–**7** Bright-field and Hoechst fluorescence images of Caco-2 tumors; the angular shape of certain tumors is indicated by thick white arrows. Apparent central hollowness of tumors in islands 1, 2, 6 and 7 that are characteristic of glandular-like adenocarcinoma are indicated by an asterisk in Hoechst images. Secondary tumor formation possibly linked to cell migration is visible on one image of each set of images for MCF7 (#xii) and Caco-2 (#2) tumors, as shown by blue arrows.

To determine the extent for the single cell culture on collagen I-island approach to recapitulate the characteristics of different types of cancer, we also tested the method with Caco-2 cells that represent a poorly aggressive colorectal carcinoma. Bright-field images obtained on day 10 of culture show that only 30% of the single cells led to tumor formation. Moreover, the rate of tumor growth seemed slower in comparison to the MCF-7 cells for the same ECM conditions, as illustrated by the smaller area of the tumors (Fig. 3, Supplementary Fig. S5); although it is noteworthy that these cultures were not done in parallel with those of MCF7 cells. Importantly, for both MCF7 and Caco-2 cells, the sizes and shapes of tumors varied considerably. Arm-like extensions often reveal the invasive nature of tumors^43, 44^. These features were very rarely observed, but one tumor (out of 15 for MCF7 cells and out of six for Caco-2 cells) for each cancer type appeared fragmented (i.e., as if one or more cells migrated away from the main tumor to create a secondary tumor; see island xii in Fig. 3a and island 2 in Fig. 3b). Tumors produced by individual Caco-2 cells looked different from tumors produced by individual MCF7 cells. For most Caco-2 tumors, remarkable features included more angular shapes and hollow structures (islands 1, 2, 6, 7) reminiscent of glandular-like adenocarcinoma seen in vivo^45^ (Fig. 3b). Interestingly, highly aggressive MDA-MB-231 cells that represent triple negative breast cancer subtype, did not form tumors (i.e. an actual growth into a mass). Instead, the cells proliferated, but moved away from each other while remaining attached to the matrix island (Supplementary Fig. S4). The aggressive nature of these cells was expressed via their migratory behavior and spindle-like shape revealing a mesenchymal phenotype).

In light of the apparent heterogeneity in tumor development (which was illustrated by tumors growing into different sizes and shapes over the same time period), a quantitative assessment was conducted with MCF7 tumors. Three morphometric parameters, namely area, circularity (ratio of area to the square of the perimeter scaled by a factor of 4π) and aspect ratio (ratio of major axis to minor axis), were analyzed with Image J^46^. The standard deviation in the parameters was relatively high in all experiments as shown for three replicates of MCF-7 cells. Even when tumors were generated from single cells coming from the same batch and passage of the cell line, and cultured under the same microenvironmental conditions, they differed strikingly from one another in terms of morphometry, revealing intertumor heterogeneity (Supplementary Fig. S5a; see also Fig. 2b). Similar results were observed with Caco-2 cells (Supplementary Fig. S5b). Only moderately negative (Circularity vs. Aspect Ratio: Pearson Correlation Coefficient: − 0.66) or no correlation (Pearson Correlation Coefficient: − 0.14 and 0.016 for Tumor Area vs. Aspect Ratio and Tumor Area vs. Circularity, respectively) was observed between any two of the three parameters in MCF7 tumors. Moreover, the depth of MCF-7 tumors measured after 14 Days in culture using confocal microscopy, was on average 75 µm in the z-dimension with a standard deviation of 19.7 µm, suggesting that there is significant variation in the number of layers of cells (a cell is on average ~ 15 µm in size) for the structures produced on the islands of collagen I.

The degree of heterogeneity in MCF7 tumor sizes was paralleled by heterogeneity in response to Paclitaxel, a commonly used cytotoxic drug for the treatment of breast invasive ductal carcinoma, for which luminal A breast cancers may show different levels of sensitivity depending on the tumor phenotype^47^. Thirteen-day old tumors were treated with three different concentrations of Paclitaxel (5 nM, 20 nM and 100 nM) for 24 h. The control was a separate dish containing islands seeded with single cells from the same population as the treated dishes and cultured with the same preparation of medium, to which 0.01% DMSO was added in order to match the maximum concentration of vehicle used in drug-treated samples. It is widely known that cells undergoing apoptosis exhibit a distinctive nuclear morphology as compared to healthy and necrotic cells^48^. Hence, tumors were treated with Hoechst 33342 nuclear dye to calculate the percentage of cells undergoing apoptosis based on nuclear morphology. Since each island comprises a relatively small amount of collagen (e.g. as opposed to an entire well of a 96-well plate), excess dye that inadvertently diffuses into the collagen can be washed easily to reduce any background fluorescence signal. Live and dead cells (the latter indicated by smaller or fragmented nuclei with intense staining due to DNA condensation) from individual planes of z-stack confocal images of each tumor were manually counted according to a procedure described by Crowley et al.^48^. As expected, the average cell death increased with drug concentration, starting with cell death at 2.9% for the control sample (Supplementary Fig. S6). Interestingly, there was a significant variation in the response of individual tumors for each drug concentration, confirming intertumor heterogeneity. In conclusion, although tumors from the cell population display some of the main characteristics of the type of cancer from which they originated, there are significant phenotypic variations among tumors produced by cells that are individually picked from this population.

### Tumor size positively correlates with intratumor heterogeneity and nuclear size in luminal A type of breast cancer

**Figure 4:**
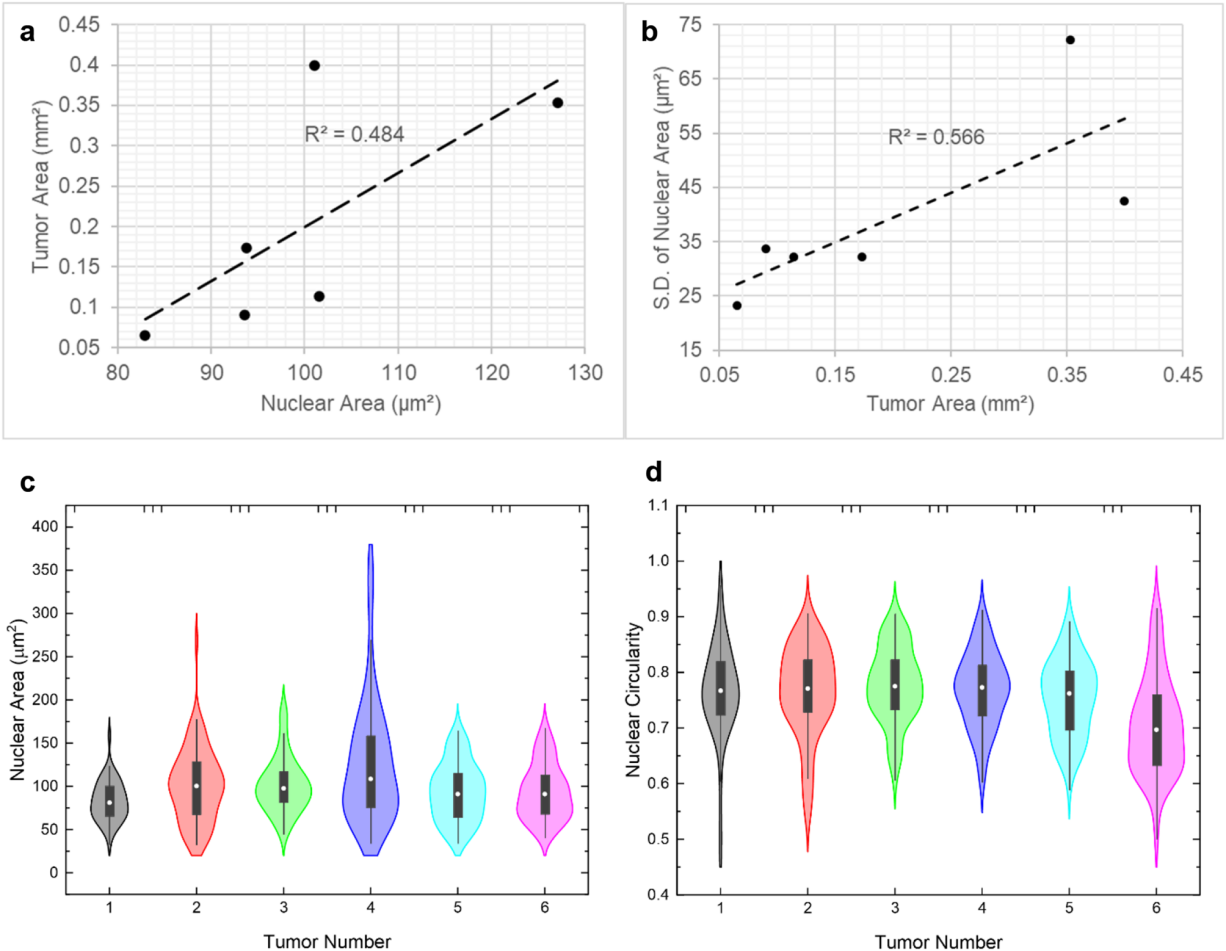
Morphometry analysis reveals a positive correlation between tumor area and high average as well as heterogeneity in nuclear area. Single MCF7 cells were cultured on collagen I islands, as described in Fig. 1. At day 14 of culture, area and circularity of tumors and areas of nuclei were analyzed with ImageJ. Trendline and associated R-square for the fit are shown with high positive Pearson correlation (r ≥ 0.70, n = 6) between nuclear area and tumor area (**a**) and between tumor area and standard deviation [S.D.] in nuclear area (used as a measure of heterogeneity) (**b**). **c** and **d** Violin plots of nuclear area and circularity for each of the six tumors where 50–75 nuclei were analyzed per tumor.

Results related to tumor size and shape presented above deserved a particular attention in terms of possible new biological information. We investigated whether the variation in tumor size and shape formed by individual cells from the same initial population might be an indication of phenotypic heterogeneity. Pathologists have relied on simple nuclear morphometric features such as size (or area) and shape (or circularity) for decades to determine tumor aggressiveness. We have also applied this method to assess tumor progression in 3D cell culture of preinvasive breast cancer cells^49^. The nuclei from six MCF-7 tumors at day 14 of culture, from the same biological replicate (i.e., seeding cells were from the same cell culture flask), were stained with Hoechst and imaged by confocal microscopy (Supplementary Fig. S7). Analysis was performed with Fiji^50^ for nuclear area and circularity and was compared to the area and circularity of the corresponding tumors. Based on 50–75 randomly selected nuclei for each tumor, there was a significantly positive correlation (Pearson’s coefficient correlation r ≥ 0.70) between tumor area and the average nuclear area (i.e., the bigger the tumor, the bigger the nuclei on average in that tumor) (Fig. 4a). We also compared the same morphometric parameters (circularity and area) for each tumor with the standard deviation of the circularity and area of their nuclei, since intratumor heterogeneity has been associated with changes in phenotype^51^. There was a significant positive correlation (Pearson’s correlation coefficient, r ≥ 0.70) between tumor area and the degree of heterogeneity in nuclear area (Fig. 4b). There was no significant correlation between any other two parameters (i.e. Pearson’s coefficient < 0.7) (Supplementary Fig. S8). The trends in nuclear area and circularity for each tumor are observed in more detail in violin plots (Fig. 4c, d). The two tumors with the highest variation in nuclear area (tumors 2 and 4) have a narrow tail, indicating that a small fraction of the nuclei have significantly larger nuclear area. As can be noted from Supplementary Fig. S7b, these particular tumors also have the largest area (0.4 and 0.35 mm^2^). Hence, this small population of cells might indeed have a causal relationship with large tumor area and may be the chief driver of tumor aggression. Heterogeneity in nuclear area does not always correspond to heterogeneity in nuclear circularity. Higher heterogeneity for nuclear circularity exists in tumors 1, 2 and 6. Especially in tumors 2 and 6, we observe wider distribution of nuclear circularity around the mean of the violin plots indicating amplified heterogeneity. It is possible that the observed differences in intratumor heterogeneity influence the variation in tumor area as seen in Fig. 2b.

**Figure 5:**
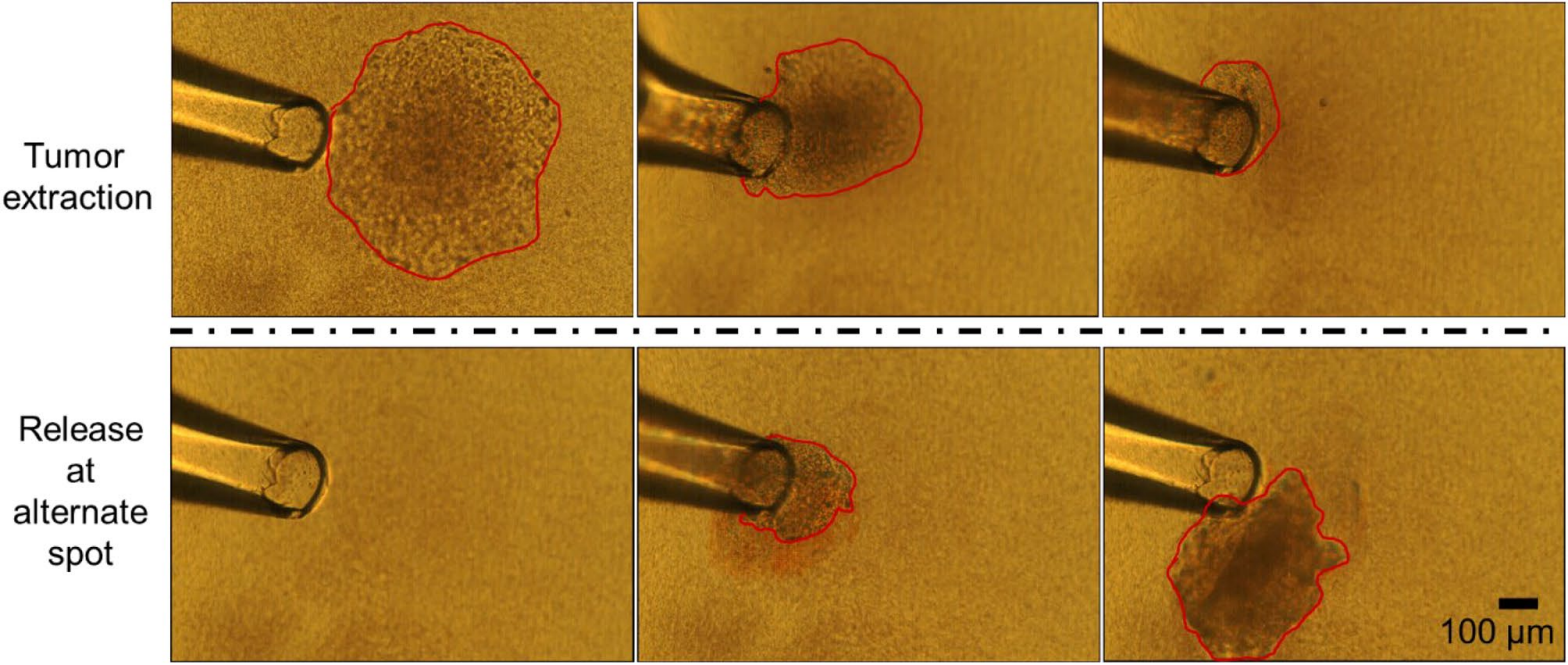
A tumor on top of the collagen I island can be easily extracted. Top: Successive images of a tumor being extracted from a collagen island, after 20–30 min treatment with collagenase, using a glass tip of ~ 150 µm diameter; Bottom: Successive images of the same tumor being released at an alternate location (e.g., for applications such as dissociation and re-seeding of selected single cells from the tumor). The red outline delineates the visible portion of the tumor during different steps of the process. (see video in Supplementary movie).

An additional advantage of culturing tumors on top of collagen islands (instead of inside) is the simplicity of tumor retrieval for further analysis (Fig. 5; Supplementary movie). To determine whether intratumor heterogeneity revealed by nuclear morphometry analysis corresponds to the presence of different cell phenotypes, a 14-day tumor formed by one MCF7 cell was released from the matrix using collagenase and cell/tumor picking setup for transfer (see supplementary Fig. S9). After separation with Trypsin–EDTA, 32 single cells were picked and seeded on new collagen islands. At day 10 of culture, 24 of the islands displayed cells. Measurement of heterogeneity based on size and shape of these second-generation tumors showed high variability in three morphometric parameters (average area: 0.03 mm^2^ with standard deviation (S.D.) 0.023 mm^2^, average circularity: 0.49 with S.D. 0.18, and average aspect ratio: 1.75 with S.D. 0.72). Thus, intratumor heterogeneity in the first-generation tumor was associated with the formation of second-generation tumors with varied sizes and shapes (Supplementary Fig. S10).

## Discussion

We have developed a highly efficient and effective method to generate tumors from isolated, individually targeted single cells. Our protocol achieves this goal in a deterministic manner by physically relocating each individual cell from its source to the top of a matrix island. Morphometric analysis of the tumors produced by single cancer cells revealed both intertumor and intratumor heterogeneity. Notably, comparison of morphometric parameters between tumors and their nuclei demonstrated a positive link between tumor size in luminal A breast cancers and the size of nucleus (based on its average and its variability).

For the purpose of preliminary demonstration of the utility of the approach, we cultured single cells from MCF-7 (poorly aggressive luminal A) and MDA-MB-231 (highly aggressive, triple negative) breast cancers and from Caco-2 (poorly aggressive) colorectal cancer. Both breast and colorectal poorly aggressive lines gave rise to tumors with morphologies reminiscent of their cancer subtypes, without striking signs of invasion or migration for the great majority of tumors. The success of tumor formation was lower with Caco-2 cells compared to MCF7 cells, possibly because there is a smaller proportion of cells with active proliferation capabilities in the Caco-2 population. This interesting observation would otherwise not be detectable had the culture been started using many cells, instead of individual cells (indeed a high proportion of cells in G0- or out of the cell cycle, might be present in cancers^52^, until a specific stimulus ‘wakes them up’). In that regards, colorectal cancer cells appear to be sensitive to mechanotransduction (which is in part linked to ECM stiffness) for their quiescence/proliferation switch^53^. Future studies should explore the impact of matrix stiffness and composition on tumor development depending on the phenotype of the seeded cell, since ECM composition is an important variable of colorectal cancer behavior^54^. Surprisingly, none of the MDA-MB-231 individual cells formed a tumor mass. Instead, they expressed their aggressive nature via a migratory phenotype. It needs to be emphasized that it has not been possible until now to study which specific cell phenotype among the MDA-MB-231 population is driving this aggressive behavior. Hence, it would be interesting to address this difference in behavior from a cell heterogeneity standpoint in future deterministic 3D culturing studies.

We have focused part of the analysis on intertumor heterogeneity that manifested in the form of variations in tumor morphometry, which is likely a reflection of the phenotypic heterogeneity in the initial population of cancer cells. Most interestingly, the fact that heterogeneity increased over time, as shown for tumor size, might be not only linked to the disparity in phenotype of the seeding cells but also to acquired intratumor heterogeneity. The latter was measured via nuclear morphometry, a resilient feature of changes in phenotypes^55^. Although the sample size was small, the fact that there was statistical significance when comparing tumor and nuclear morphometric parameters suggests a strong correlation between tumor architectural (size, shape) and nuclear structural features. The average nuclear area was observed to be higher in luminal A breast tumors of large size. An increase in nuclear area and larger tumors have both been considered signs of cancer aggressiveness. Moreover, tumors of large size had increased intratumor heterogeneity based on nuclear size. Intratumor heterogeneity has been considered a driver of cancer progression^51^.

We demonstrated that intratumor heterogeneity could develop from one single cell, as measured by heterogeneity in nucleus area and also, in tumor size and shape following second-generation deterministic 3D culturing, although there was no intentional selective pressure. How heterogeneity is established starting from a single cell might be linked to heterogeneity in the microenvironment (with different concentrations of signaling factors for instance). We cannot rule out such microenvironmental heterogeneity coming from neighboring matrix islands in the culture dish; however, the distance between islands (5 mm, center to center) is likely too high to produce heterogeneity linked to paracrine stimulation. Acquired heterogeneity might be the result of the intrinsic instability of cancer cells with the introduction of DNA damage and the uneven distribution of genetic material during cell division^56^. An additional possibility that the deterministic 3D culturing might help address is epigenetic heterogeneity (e.g., based on the type and quantity of epigenetic marks present at each given gene), since similarly to stem cells, many cancer cells possess marks of unstable local epigenetic make-up^57^. Upon cell division the unstable epigenetic make-up might be driven towards changes in gene expression that differ between the two daughter cells, possibly due to changes in mechanical stimuli that might depend on the distance of the cell compared to the matrix contact. We determined that nuclear area was a source of intratumor heterogeneity. However, how the size of the nucleus or its roundness may be linked to the epigenetic expression of heterogeneity remains to be identified. This result calls for further analysis of the involvement of proteins that might regulate nuclear size via a possible impact on chromatin compaction, hence influencing both nuclear morphometry and gene expression profiles.

Our approach is cost-efficient, utilizes standard laboratory equipment and consumes low quantities of reagents. Due to the discrete, island-like nature of the collagen support, a cell culture dish of 35 mm uses less than 250 µl collagen matrix and 2 ml of culture medium. This condition eliminates the need to frequently change the medium due to extremely low consumption of nutrients by the tumors. The method is highly amenable for future automation for higher speed, throughput and repeatability both for deposition of collagen islands and for choosing and seeding individual cells. Analyses such as immunomarker based assays, imaging assays, genetic sequencing assays can potentially be performed on the tumors cultured using this method (Supplementary Fig. S11).

Future work will involve selecting the seed cells based on genotypical and/or phenotypical parameters, including expression of surface markers as well as imaging of epigenetic modifications, and studying tissue development under more complex 3D cell culture conditions. Additionally, we aim to enhance the throughput of the method by using a larger number of islands as well as automating the individual cell picking and seeding process. Other potential future developments of the method will involve co-culturing by seeding a cell along with a different type of single cell. We anticipate the deterministic 3D culturing to be highly useful for a wide range of applications, including propagating extremely rare cells (like circulating tumor cells-CTCs and circulating fetal cells), as well as studying the nature and impact of phenotypic heterogeneity in tumors and tissues.

## Materials and methods

### Cell line, culture medium and treatment

MCF-7 cells (American Type Cell Culture, Manassas, VA) were cultured in RPMI 1640 medium (ATCC) with 10% Fetal Bovine Serum (ATCC). Caco-2 cells (a kind gift from Dr. Mohit Verma) were cultured with DMEM medium (ATCC) with 20% Fetal Bovine Serum and other additives (Final concentrations: 4.5 g/L Glucose, 10 mM HEPES, 44 mM Sodium Bicarbonate, 1 mM Sodium Pyruvate, 100 µM non-essential amino acids, 50 mg/L Gentamycin Sulfate, 100 U/L Penicillin, 100 U/L Streptomycin, 2 mM Glutamine, pH 7.2). MDA-MB-231 cells (ATCC) were also cultured in RPMI 1640 medium (ATCC) with 10% Fetal Bovine Serum. Paclitaxel (Selleck Chemicals, Houston, TX) prepared in DMSO (ATCC) was used to treat some of the tumors formed by single cells seeded on collagen I islands.

### Extracellular matrix

We used the PHOTOCOL UV (Advanced BioMatrix, San Diego, CA) that is a Methacrylated Type I collagen. Depending on the desired volume, and stiffness of 3,320 Pa, the lyophilized and Methacrylated Type I collagen was mixed with an appropriate amount of 20 mM acetic acid solution and a neutralization solution, as per the manufacturer’s protocol. The mixture was kept on ice to maintain the final solution in liquid state. Drops (5 µl) were added on a culture dish and used for single cell culture as described below. The thermosetting nature of PHOTOCOL helped keep the gel islands fixed onto the culture dish once they were deposited, and the dish was placed in a temperature-controlled incubator.

### Cell picking device

A cell picking device was assembled using a glass capillary micropipette tip (Clunbury Scientific LLC, Bloomfield Hills, MI) a 3-axis translational stage for accurate movement of the pipette tip (Thorlabs Inc., Newton, NJ) and 250 µl airtight SGE glass syringe with Luer Lock (Fig. 1c). A hollow glass capillary of 50 µm diameter worked as a micropipette. It was attached to an adapter, which in turn was attached to a syringe via Polytetrafluoroethylene (PTFE) tubing. The syringe was also fitted with a unidirectional translation stage for precise control of flow (subsystems extensively imaged and presented in Supplementary Fig. S9). Alternatively, the syringe can also be operated with a syringe pump. The system was optimized to manually pick up and dispense nanoliter volumes of liquid and work with an efficiency of one to two cells/min while transferring a single cell from a dish to a matrix island.

### Single cell culture seeding and maintenance

For single cell culturing, approximately 5,000 cells from the flask were obtained by dilution and placed in a dish for picking. The cells that ended up on each island were picked from among these 5,000 cells. The same culture medium mixed with 1% v/v GIBCO Penicillin–Streptomycin (10,000 U/ml) antibiotic (Life Technologies, Carlsbad, CA) was used to culture single cells. The antibiotic was added to avoid bacterial contamination that can occur when transferring cells onto the matrix island in nonsterile conditions. Five µl liquid drops of collagen matrix were deposited at desired number of spots (up to 50) on a sterile 35 mm polystyrene culture dish (CORNING 430558). The matrix was allowed to solidify for 30–45 min in the same incubator that is used for cell culture (maintained at 37 °C temperature, 5% CO_2_ concentration and 95% Relative Humidity). After stiffening of the matrix, 2 ml of RPMI 1640 culture medium premixed with 10% v/v fetal bovine serum and 1% v/v GIBCO Penicillin–Streptomycin (10,000 U/ml) antibiotic, pre-heated to 37 °C was introduced in the dish. This was done slowly by placing the pipette tip against the side of the dish to avoid air bubbles and dislocation of the islands. Then the dish was placed into the incubator until cells were ready for seeding. Addition of cell culture medium allowed for the matrix to remain solid without drying out. When it was time to seed the cells, 1.8 ml of culture medium was removed from side of the dish, which exposed the top of each island while keeping the islands partially immersed to avoid drying.

The cell-picking setup was used to pick and place single cells from another dish to the top of Collagen I islands one at a time, using a microscope. After all the cells were transferred, the dish was covered and placed in the cell culture incubator for 20–30 min to allow the cells to stick to the matrix. During this period the surrounding liquid medium at the bottom of the dish provided enough moisture to avoid drying. At the end of this period of time, 1.8 ml culture medium with 1% v/v antibiotic was replenished gently, and the culture dish was returned into the incubator.

The culture medium was changed every 5–7 days initially with single cells and when the tumors were small, and more frequently (every 2–3 days) as tumors became larger. Removing and adding medium was performed from the side of the culture dish to avoid turbulence.

### Hoechst staining and microscopy

Cells were washed in PBS and fixed in 4% paraformaldehyde before staining with 1:1,000 PBS diluted solution of 20 mM Hoechst 33342 (Thermo Fischer Scientific, Waltham, MA) for 10 min. After washing once with PBS, tumors were imaged with a ZEISS LSM 800 confocal microscope with an excitation laser of 401 nm wavelength and with 10X magnification lens (EC Plan-Neofluar 10X/0.30 M27). Bright-field and fluorescence images were also obtained using NIKON ECLIPSE 80i upright fluorescence microscope, which were used to analyze the morphology of the tumors with ImageJ. Z-stacks were obtained for each tumor at a Z-step size equal to half of the depth of field which was 14.5 µm (using objective: EC Plan-Neofluar 10x/0.30 M27). For each tumor, dead and live nuclei at every alternate plane of the z-stack were manually counted using Fiji. Each alternate plane of the Z-stack was skipped to account for the overlap in signal due to depth of field being twice that of Z-step size. This approach allowed for better resolution images and prevented any double counting of cells. A similar approach was used for the assessment of nuclear morphometry after manual segmentation with Fiji.

### Retrieval of tumors

The 3D cultures were treated with collagenase for 20–30 min to partially digest the surface of the collagen islands and help detach tumors from the top of the island. A pipette tip of ~ 150 µm diameter was used for retrieval of tumors of 0.1–0.2 mm^2^ in size on average, under a microscope. The diameter of the tip was chosen so that it is significantly smaller than the island but similar in size to the tumor in order to retrieve the tumor without accidentally aspirating an entire collagen island, as shown in the Supplementary Movie. A larger diameter of the tip may be used depending on the size of tumors. Manual control of the picking process permitted more precision for the retrieval of tumors, without breaking them apart.

### Re-culturing of single cells from a tumor

A desired tumor was extracted as described before. Then, 10 µl of Trypsin–EDTA solution was placed in the cap of a small RNA-free tube. The extracted tumor was introduced into the trypsin solution for 5 min. The rest of the tube was filled with 100 µl of culture medium with 10% FBS and the tube was centrifuged gently (600 g for 5 s) to mix the Trypsin with the culture medium and stop Trypsin activity. The liquid was moved up and down rapidly with a 5 µl pipette to break down the tumor into smaller clusters. All of the liquid was removed and placed in the center of a 35 mm culture dish. Another 500 µl of culture medium was added gently and single cells were picked up as described before.

### Statistical analyses

To analyze the variation in nuclear area and nuclear circularity for heterogeneity analysis, the sample standard deviation formula in MS EXCEL was used. Pearson Correlation coefficient function of MS EXCEL was used to study correlation between every two of the following six parameters: Tumor area, tumor circularity, Average Nuclear Area, Average Nuclear Circularity, Standard Deviation of Nuclear Area, Standard Deviation of Nuclear Circularity. Violin plots for nuclear area and nuclear circularity were created using the ORIGIN(Academic), Version 2019b, OriginLab Corporation, Northampton, MA.

A two-tailed heteroscedastic (unequal variance) T-test was used to compare the percentages of cell death between tumors treated with different concentrations of Paclitaxel (comparisons were made between 0 and 5 nM; 5 nM and 20 nM; 20 nM and 100 nM). A similar test was used to check statistical difference among the three replicates for comparing tumor morphologies.

## Supplementary information

Supplementary Information

Supplementary Movie

## Supplementary information

is available for this article at 10.1038/s41598-020-67674-3.
