# Supplementary material for: Deterministic culturing of single cells in 3D
Source: Sci Rep. 2020 Jul 2;10:10805. doi: 10.1038/s41598-020-67674-3 (PMC7331589; doi:10.1038/s41598-020-67674-3)
Supplement: Supplementary file 1 — Supplementary Information [file 41598_2020_67674_MOESM1_ESM.pdf]

# Supplementary Information

# Deterministic Culturing of Single Cells in 3D

Rohil Jain[a,b], Shirisha Chittiboyina[b,d], Chun-Li Chang[a,b], Sophie A. Lelièvre*[b,d,e], Cagri A. Savran*[a,b,c,e]

[a]School of Mechanical Engineering, Purdue University, West Lafayette, IN, 47907, USA

[b]Birck Nanotechnology Center, Purdue University, West Lafayette, IN, 47907, USA

[c]Weldon School of Biomedical Engineering, Purdue University, West Lafayette, IN, 47907, USA

[d]Department of Basic Medical Sciences, College of Veterinary Medicine, Purdue University, West Lafayette, IN, 47907, USA

[e]Center for Cancer Research, Purdue University, West Lafayette, IN, 47907, USA

*Corresponding Authors, Email: savran@purdue.edu, lelievre@purdue.edu

**<u>Supplementary S1</u>: Isolated-cell Culture Characteristics**

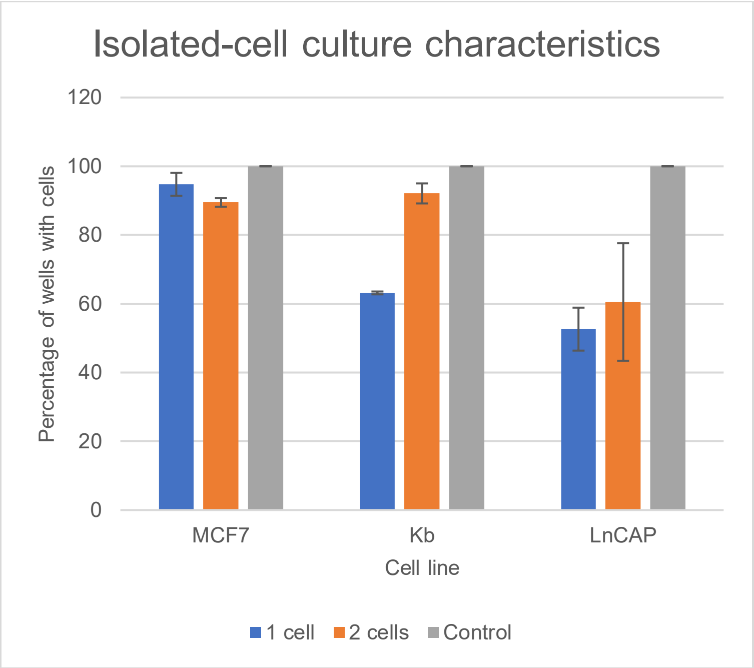


Cells from Cervical Cancer (KB line), Prostate Cancer (LNCaP line) and Breast Cancer (MCF-7 line) were seeded in 96 wells on plastic (2D culture), either as single cells (blue bars), two cells (orange bars) or 100 cells (gray bars, control). Show on the bar graph are percentages of wells with cells after 10 days in culture. Average presented with standard deviation (n = 2).

**<u>Supplementary S2</u>: Figure 2a without the markings for better visualization of the tumors**

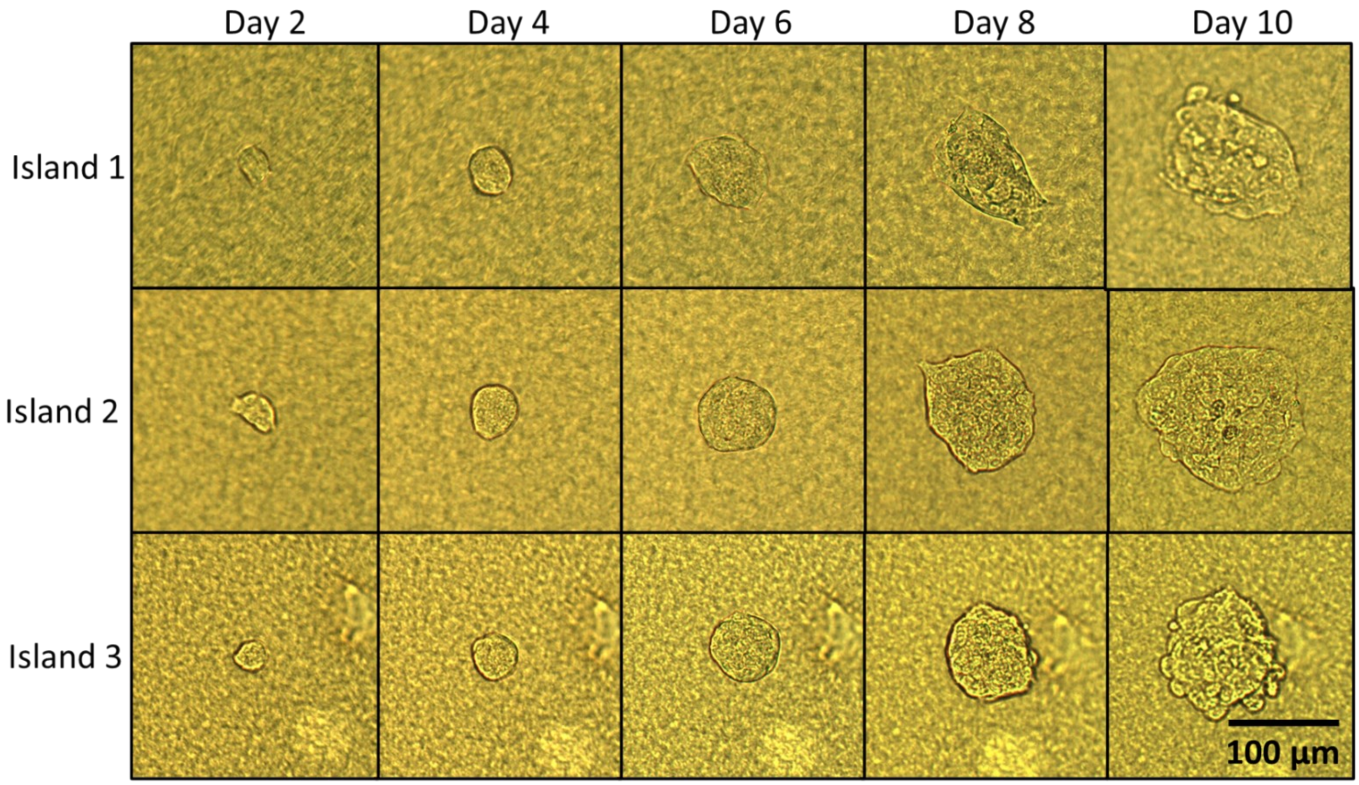

**<u>Supplementary S3:</u> Observation of necrotic core in long-term cultures**

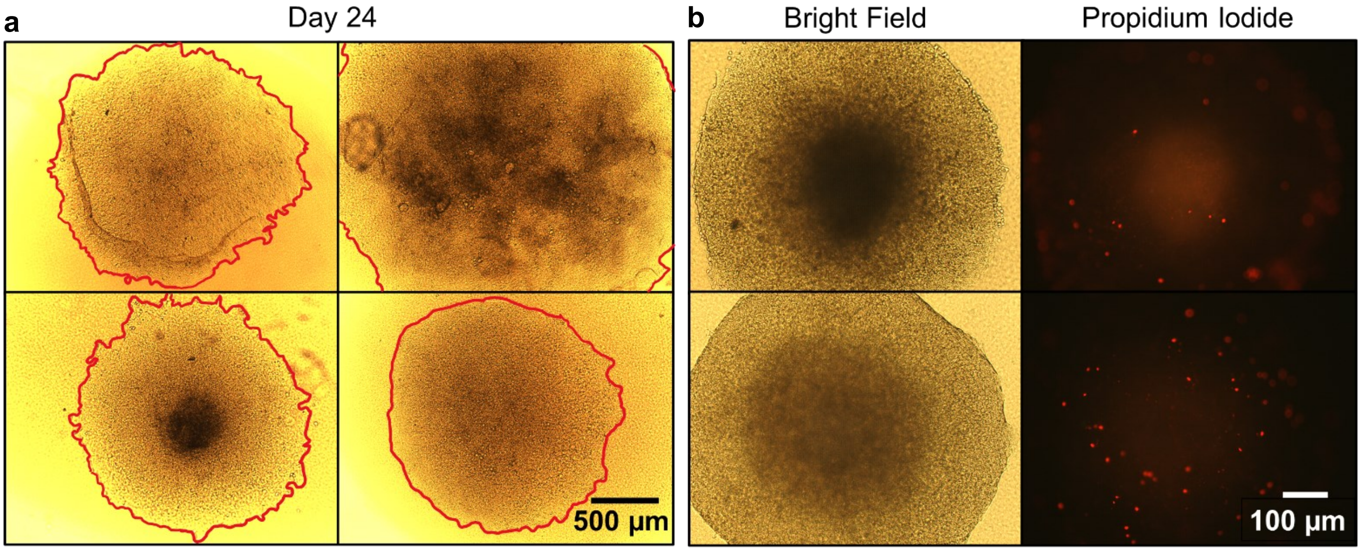


Single MCF7 cells were cultured on collagen islands for 24 to 26 days. **a,** Images of four tumors after 24 days of culture (with tumors delineated in red) **b,** Images of two tumors from another experiment after 26 days of culture that display darker central regions, as recorded with bright-field microscopy. Propidium Iodide (orange diffuse staining) images of necrosis are shown for the two tumors. Such darker central areas are observed in less than 8% of tumors that are cultured for long periods of time (more than 21 Days).

**<u>Supplementary S4:</u> MDA-MB-231 cell behavior on collagen I islands**

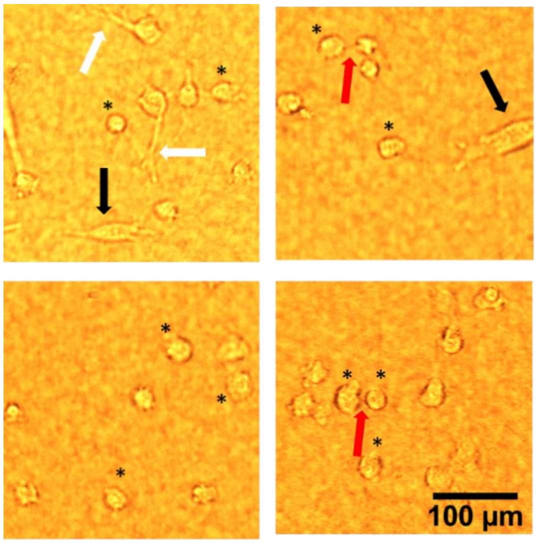


Single MDA-MB-231 cells were cultured on collagen islands for 10 days. In four representative islands some of the individual cells resulting from proliferation are indicated by an asterisk (rounded cells are either still, dying or undergoing mitosis). Some of the cells display a pseudopodia-like extension that may indicate migratory activity (see white arrows), which gives a possible explanation for the distance observed between cells on an island, and other cells display spindle-like shape (black arrow) characteristic of aggressive breast carcinomas that contain a mesenchymal type of cells. Cell division may lead to daughter cells of uneven size (red arrows).

**<u>Supplementary S5:</u> Distribution of morphological characteristics of tumors generated by single MCF-7 and Caco-2 cells.**

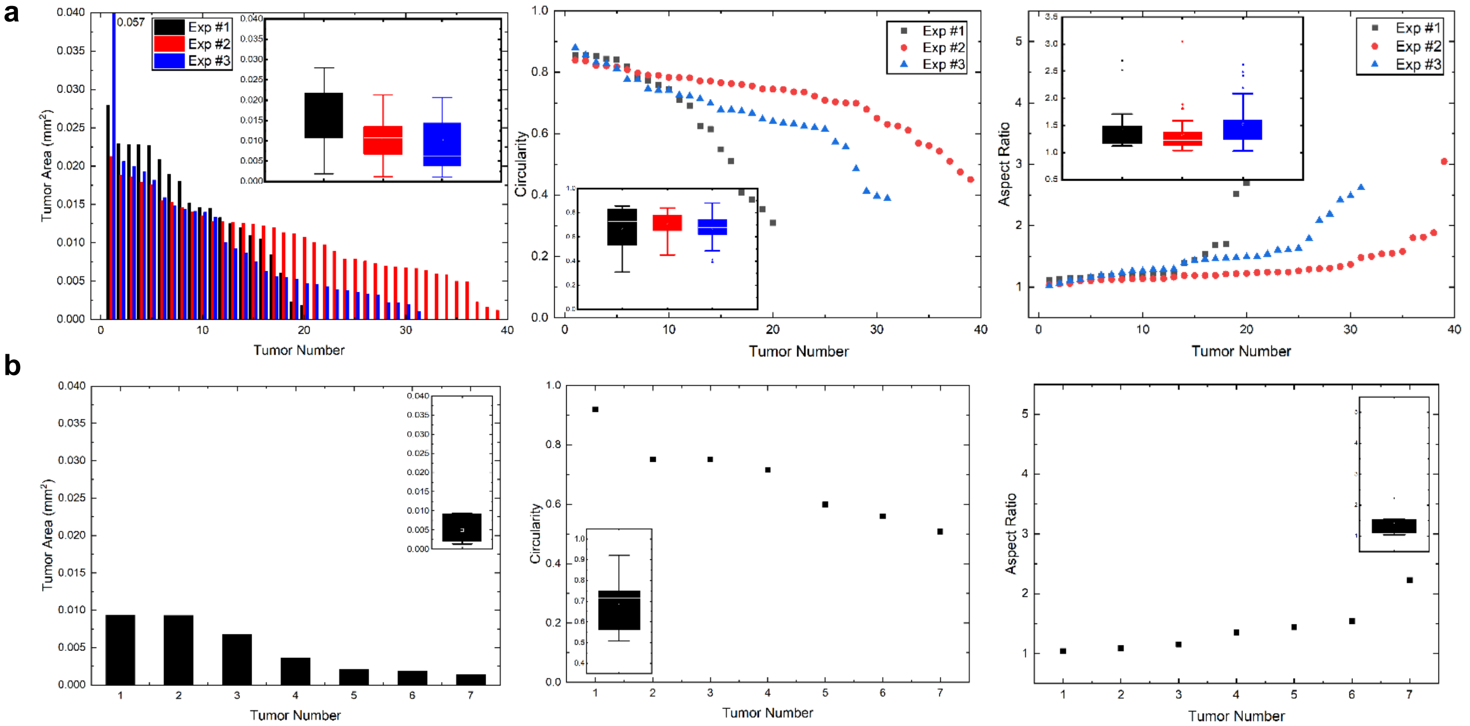


Single MCF7 and Caco-2 cells were cultured on collagen islands for 9-10 days. **a,** Quantitative assessment of morphometry (area, circularity, aspect ratio) in three replicates (Exp. #1, 2 and 3) of MCF7 single cell cultures that gave rise to 26 to 47 tumors. Inset in all graphs represents the box plot for each experiment. **b,** Morphometry analysis of the tumors developed by single Caco-2 cells (one replicate). Insets show box plot for the data.

**<u>Supplementary S6:</u> Treatment of MCF7 tumors with paclitaxel**

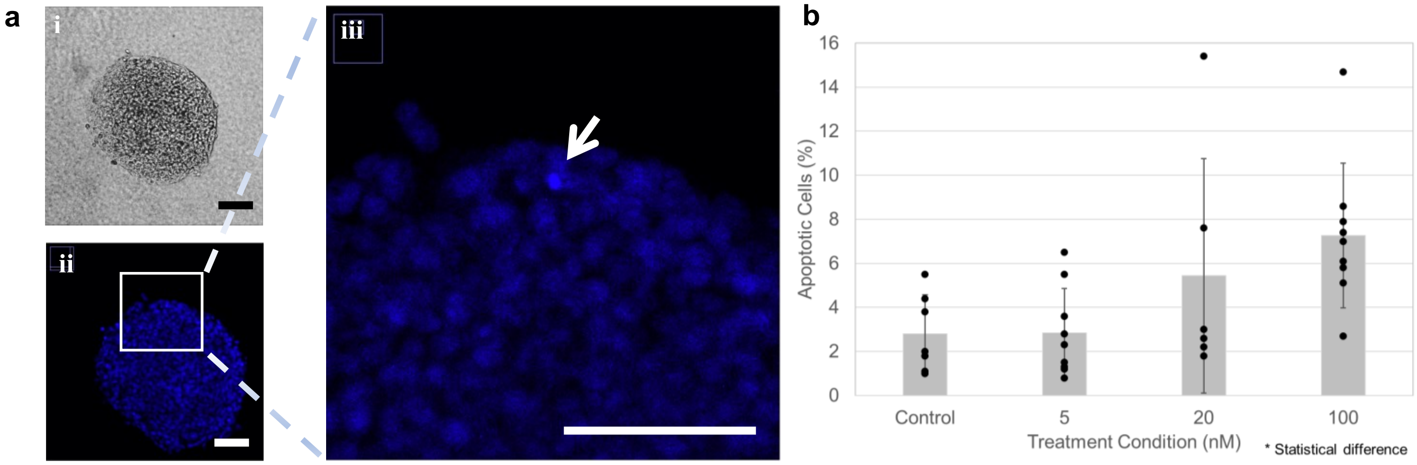


Single MCF7 cells were cultured on collagen islands for 13 days before treatment with paclitaxel or vehicle DMSO (Control) for 24 hours. **a: i & ii,** Bright-field and confocal fluorescence (Hoechst) images of a tumor after treatment with 5 nM of paclitaxel. **iii** Zoomed portion of image ii showing nuclei (in blue) with one apoptotic (smaller and brighter) nucleus (white arrow). Scale bar: 100 µm. **b,** Bar graph of the percentage of apoptotic cells. Black dots represent individual tumors and black vertical lines represent standard deviations.

Two-tailed heteroscedastic t-test based P-values for each of the six pairs of treatment are as follows: DMSO with 5 nM-0.972, DMSO with 20 nM-0.291, DMSO with 100 nM-0.004, 5 nM with 20 nM-0.297, 5 nM with 100 nM-0.004, 20 nM with 100 nM-0.477.

**Supplementary S7: Images and tumor area for the six tumors used for heterogeneity analysis.**

a

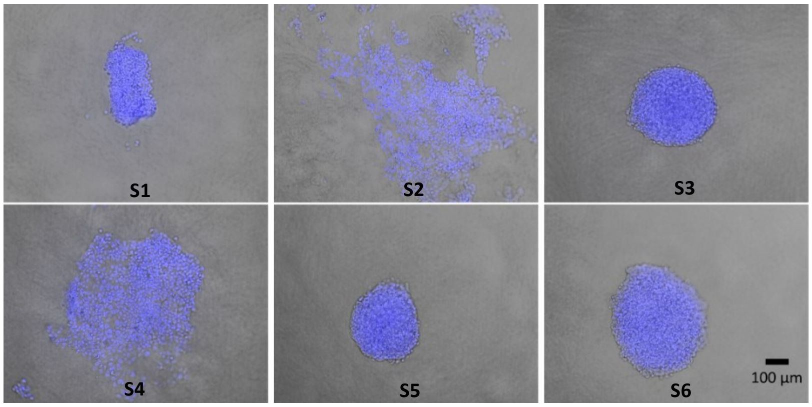


b

| Tumor number | 1 | 2 | 3 | 4 | 5 | 6 |
|---|---|---|---|---|---|---|
| Area ($mm^2$) | 0.065 | 0.40 | 0.114 | 0.353 | 0.091 | 0.173 |
| Circularity | 0.547 | 0.233 | 0.81 | 0.636 | 0.767 | 0.75 |

Single MCF7 cells were cultured on collagen islands for 14 days. **a,** Merged images of six tumors (S1 to S6) recorded using bright-field and fluorescence imaging (fluorescence microscope, Nikon ECLIPSE 8i) following Hoechst staining that were used to obtain tumor morphology information. This information combined with nuclear morphometry information obtained with the confocal microscope were used for the analysis of the potential link between parameters of tumor morphometry and nuclear morphometry (see Figure 4). **b,** Area and circularity for each tumor in the group.

**Supplementary S8: Pearson correlations between all other parameters for the six tumors in Supplementary S6.**

| | Average Nuclear Circularity | Average Nuclear Area | Tumor Circularity | Tumor Area | S.D. Nuclear Circularity | S.D. Nuclear Area |
|---|---|---|---|---|---|---|
| Average Nuclear Circularity | 1 | | | | | |
| Average Nuclear Area | 0.29 | 1 | | | | |
| Tumor Circularity | -0.26 | -0.01 | 1 | | | |
| Tumor Area | 0.13 | **0.70** | -0.66 | 1 | | |
| S.D. Nuclear Circularity | -0.58 | -0.62 | -0.39 | -0.06 | 1 | |
| S.D. Nuclear Area | 0.23 | 0.97 | -0.14 | **0.75** | -0.50 | 1 |

**Supplementary S9:** **Cell picking setup parts with magnified images.**

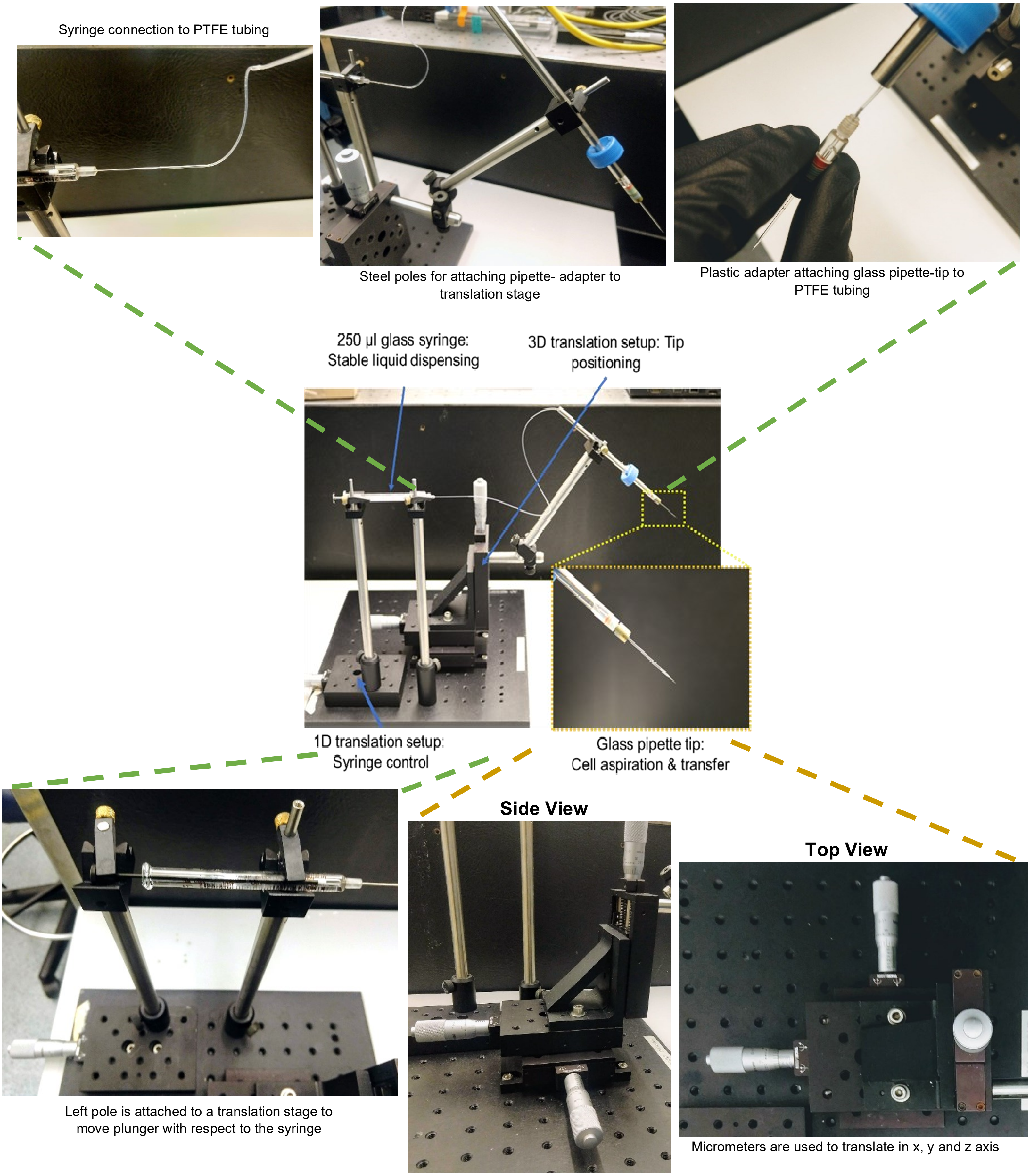

**<u>Supplementary S10</u>: Re-cultured cells forming tumors**

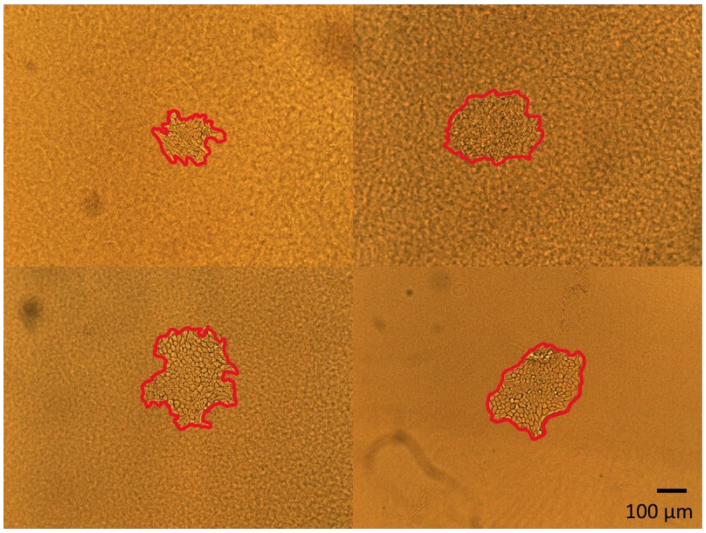


Day 10 bright field images of tumors (marked in red) obtained by re-culturing of single cells after breaking an initial tumor into single cells as described in the methods section.

**<u>Supplementary S11</u>: Future possibilities of analyses and assays that can be performed using the method**

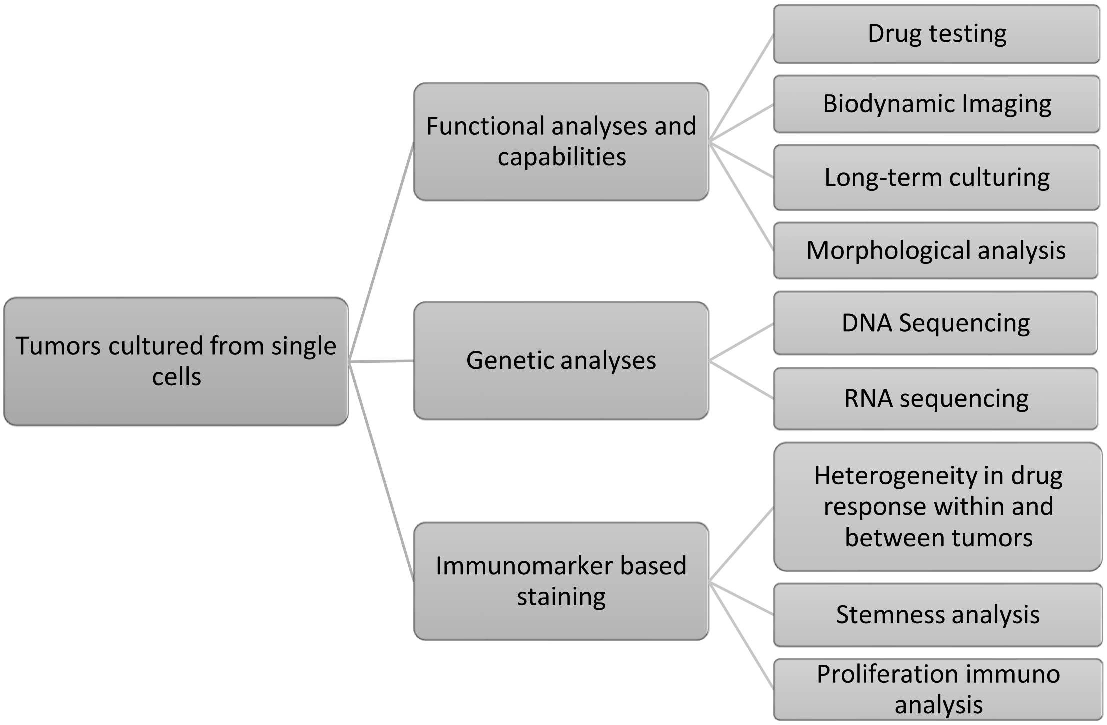

**<u>Supplementary Movie:</u> Video showing tumor pick up and retraction using a micropipette**

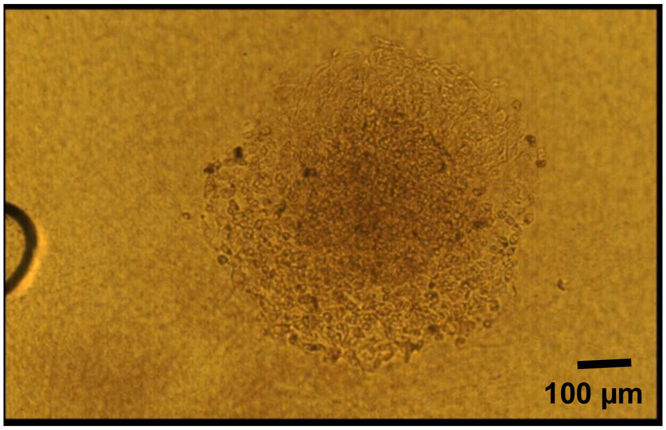


The attached video shows the process of picking up and retracting a tumor in real-time, under 10x magnification of a bright field microscope.
